# Delivery accuracy of VMAT on two beam‐matched linacs provided by accelerated go live service

**DOI:** 10.1002/acm2.14071

**Published:** 2023-06-16

**Authors:** Masato Tsuneda, Kota Abe, Yukio Fujita, Ryo Morimoto, Takuma Hashimoto, Yukinao Abe, Takashi Uno

**Affiliations:** ^1^ Department of Radiation Oncology MR Linac ART Division Graduate School of Medicine Chiba University Chuo‐ku Chiba Japan; ^2^ Department of Radiation Sciences Komazawa University Setagaya‐ku Tokyo Japan; ^3^ Department of Radiology Chiba University Hospital Chuo‐ku Chiba Japan; ^4^ Diagnostic Radiology and Radiation Oncology Graduate School of Medicine Chiba University Chuo‐ku Chiba Japan

**Keywords:** beam data collection, beam matching, commissioning, patient specific QA, VMAT

## Abstract

**Introduction:**

Dosimetric accuracy is critical when a patient treated with volumetric modulated arc therapy (VMAT) is transferred to another beam‐matched linac. To evaluate the performance of Accelerated Go Live (AGL) service, the measured beam characteristics and patient specific quality assurance (QA) results between two AGL‐matched linacs were compared.

**Materials and Methods:**

Two VersaHD linacs were installed using the AGL service. After the installation, the beam data such as percentage depth dose (PDD), lateral profiles and output factors for all photon beams were measured. Relative doses were also measured as a function of the multi‐leaf collimator (MLC) leaf gap width. Subsequently, VMAT plans were created for prostate, pelvis, head and neck, liver, lung cancers and multiple brain metastases. Dose distributions and point doses were measured by multi‐dimensional detectors and ionization chambers for patient specific quality assurance, and comparisons were made between the two linacs.

**Results:**

Dose differences in PDDs were all within ± 1% except the entrance region, and the averaged gamma indices of the lateral profiles were within 0.3. The differences in doses as a function of the MLC leaf gap width between the two linacs were within ±0.5%. For all the plans, gamma passing rates were all higher than 95% with criteria of 2%/2 mm. The average and the SD of dose differences on the multi‐dimensional detector between both measurements was 0.06 ± 2.12%, and the average of point dose differences was −0.03 ± 0.33%.

**Conclusion:**

We have evaluated the AGL performance in the context of beam characteristics and patient specific QA. It was demonstrated that the AGL service provides an accurate VMAT treatment reproducibility for many tumor sites with gamma pass rates greater than 95% under criteria of 2%/2 mm.

## 1 INTRODUCTION

Quality assurance (QA) of linear accelerators (linacs) aims to ensure that the dose distributions delivered to a patient body reproduce those planned by a treatment planning system (TPS). A beam matching of multiple linacs traditionally involves matching mechanical and beam characteristics of each linac to reference values within tolerances. For example, Varian Medical Systems provides representative beam data (RBD) for their TrueBeam linacs in the form of scanning and non‐scanning data required for beam modeling for the Eclipse TPS (Varian Medical Systems, Palo Alto, USA). This procedure presumes that any variations in the machine characteristics of the linacs are small. However, the RBD were created by averaging the beam data from three TrueBeam linacs at a single institution,^1^ without disclosing the variations of the data among the three machines. Tanaka et al. compared beam data among 21 TrueBeam linacs and showed good agreement for field sizes ≥10 × 10 cm^2^.^2^ They also demonstrated that the dose profiles showed variations larger than 2% for a field size of 3 × 3 cm^2^. Tani et al. compared beam data for 15 TrueBeam linacs and showed good agreement.^3^ Sjostrom et al. reported that it is not always possible to match linacs and some specific models might be required.^4^

Elekta has recently provided Accelerated Go Live (AGL) service.^5^ The AGL aims to directly match the measured beam data to the beam model in Monaco TPS (Elekta, Stockholm, Sweden). This beam model is provided by the vendor and the gamma analysis with a 2% dose difference (DD) and a 2‐mm distance to agreement (DTA) is employed to evaluate the agreement between the calculation and the measurement during the AGL workflow. By using the AGL service, it was reported that the beam commissioning processes among multiple Elekta Precise linacs can be significantly accelerated, thus enabling faster clinical use.^5^ However, dosimetric accuracy and variations of volumetric modulated arc therapy (VMAT) deliveries among multiple AGL‐matched Elekta linacs are still unknown.

The purpose of this study was to evaluate the AGL performance in the context of patient specific QA. In this report, firstly, measured beam data were compared between two AGL‐matched Elekta linacs; and subsequently, patient specific QAs were performed and the results were compared between the two linacs recently installed in our institution.

## 2 METHODS

### 2.1 Comparison of the measured beam data between the linacs 1 and 2

Two Versa HD linacs (Elekta, Stockholm, Sweden) were utilized for the test with photon energies of 4 MV (“4X FF”), 6 MV (“6X FF”), 10 MV (“10X FF”), 6 MV FFF, (“6X FFF”) and 10 MV FFF (“10X FFF”). A 3D water phantom, BeamSCAN (PTW, Freiburg, Germany) was employed to measure all the percent depth doses (PDDs), profiles and point doses. All the measurements were performed at a source to surface distance (SSD) of 90 cm. Field sizes for the PDDs were varied from 2 × 2 cm^2^ to 40 × 40 cm^2^ defined at a source to axis distance of 100 cm. To reduce the influence of noise at the depth of dose maximum (dmax), the average value was obtained using PDD at the dmax ± 1 mm and the PDDs were normalized to the averaged value. The profiles were measured in the inline, the crossline and the diagonal directions, at the depths of 5, 10, and 20 cm. A PTW Semiflex 3D chamber was used for all the field sizes. Out of the data acquired, the PDDs with the field sizes of 3 × 3, 5 × 5, 10 × 10 and 30 × 30 cm^2^, and the profiles with a field size of 30 × 30 cm^2^ were selected for comparisons between the two linacs in this study. Measured PDDs were compared by a criterion of a global DD. Measured profiles were compared by a gamma analysis with a 2% DD, a 2‐mm DTA and a 10% dose threshold. An in‐house software was developed using a Python language for the gamma analysis.

Output factors were measured at the depth of 10 cm with the SSD of 90 cm, and compared between the two linacs for field sizes ranging from 3 × 3 to 40 × 40 cm^2^. A reference chamber 30013 (PTW Freiburg, Germany) was used for the field sizes larger than or equal to 10 × 10 cm^2^. A Semiflex 3D chamber 31021 (PTW Freiburg, Germany) was used for the smaller fields. Measurements were normalized to the value with a field size of 10 × 10 cm^2^.

To evaluate the delivery accuracy of dynamic MLC plans, the reference chamber was inserted into a hole of an ABS phantom (R‐TECH, Tokyo, Japan) at the isocenter with a depth of 10 cm. During a dose delivery of 100 MU to the phantom with 6X FF beams, the MLC leaves moved from −50 to 50 mm on the isocenter plane with a constant leaf gap width of 5, 6, 8, 10, 12, 14, 16 and 20 mm under a collimator angle of 0 degrees. A relative percentage dose was calculated by normalizing each measured dose to that for a 10 × 10 cm^2^ open field with the identical dose delivery. The calculated relative doses on the two linacs were compared, and the percentage DDs between them were also obtained.

### 2.2 Patient specific QA comparison between the linacs 1 and 2

Clinical VMAT plans were created for prostate, pelvis, head and neck (H&N), liver, lung cancers and multiple brain metastases using two treatment planning systems (TPS), Monaco (Elekta, Stockholm, Sweden) and RayStation (Ray‐Search Laboratories, Stockholm, Sweden). The SRS MapCHECK (Sun Nuclear, Florida, USA) having a detector spacing of 2.47 mm was used for the patient specific QA of the multiple brain metastases plans, whereas the Delta4 (ScandiDos, Uppsala, Sweden) was employed for other tumor sites mentioned above. The measurements were made on the two linacs, and the gamma analyses of a 3% DD and a 2‐mm DTA with global dose normalization were performed for all the plans in accordance with the AAPM TG‐218 guideline. Besides, a more strict criterion of a 2% DD and a 2‐mm DTA was also employed. Furthermore, the 3D PinPoint chamber 31015 (PTW Freiburg, Germany) and the reference chamber were used for point dose measurements in selected phantoms. Each of the measured point doses were compared with each of the calculated doses at the identical positions.

In addition to the patient specific QA, the measurements between the two linacs were directly compared to evaluate the dosimetric reproducibility for the VMAT delivery. The SRS MapCHECK was used for the measurement of multiple brain metastases, whereas Delta4 was employed for the H&N, pelvis, prostate, lung and liver plans. The DD maps were created by subtracting the dose on the linac 2 from that on the linac 1, and normalizing by the maximum dose, where the number of points receiving greater than one‐tenth of the prescribed dose were counted to generate histograms. We calculated the average and the standard deviation of the DDs for each tumor site. The point doses measured on the linacs 1 and 2 were also compared.

## 3 RESULTS

### 3.1 Comparisons of measured beam data between the linacs 1 and 2

#### 3.1.1 PDDs and profiles

**FIGURE 1:**
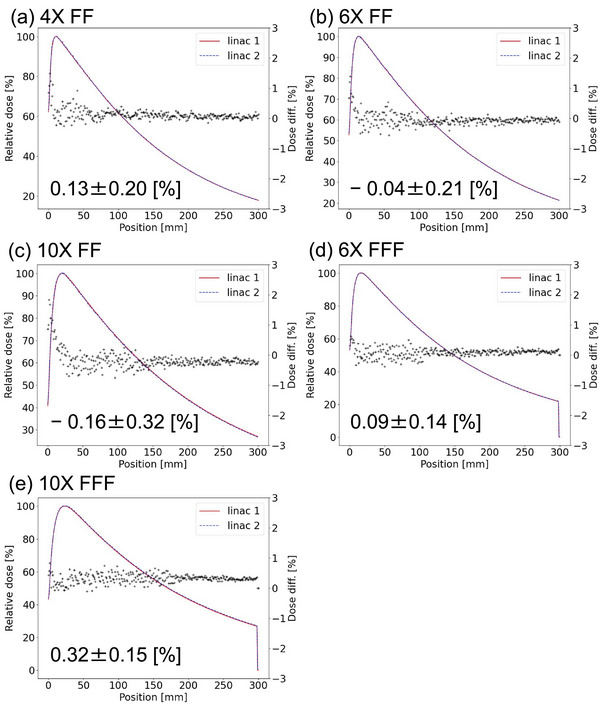
Comparisons of measured PDDs between the two linacs with a field size of 10 × 10 cm^2^ at each photon energy: (a) 4X FF, (b) 6X FF, (c) 10X FF, (d) 6X FFF, and (e) 10X FFF.

**FIGURE 2:**
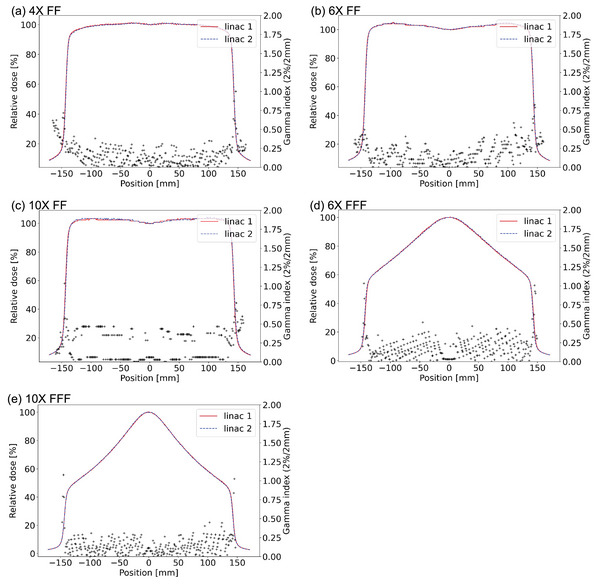
Comparisons of measured crossline profiles between the two linacs with a field size of 30 × 30 cm^2^ at each photon energy: (a) 4X FF, (b) 6X FF, (c) 10X FF, (d) 6X FFF, and (e) 10X FFF.

All the results led to the gamma pass rates of 100%, through AGL service. AGL results are described in the supplement section (Supplementary figures 1–4). We accepted the AGL‐based Monaco beam model. Figure 1a–e compares the PDDs between the two linacs with a field size of 10 × 10 cm^2^ for all the photon energies. The DDs of the PDDs between the two linacs were within 1% excepting at the entrance depths. Averaged DDs among all the measured depths were 0.13, −0.04, −0.16, 0.09, and 0.32% for 4X FF, 6X FF, 10X FF, 6X FFF, and 10X FFF, respectively. The larger errors at the shallower depths were affected by the differences in the low‐energy spectrum. We consider this difference at the surface can be clarified by comparing the PDDs of many Elekta linacs. However, these errors are not significant for the clinical use. Table 1 shows the percentage DDs of the PDDs between the two linacs for different field sizes and energies. Figure 2a–e compares the measured crossline profiles between the two linacs, showing acceptable agreements. Averaged gamma indices of the crossline and the inline profiles denoted inside the parentheses in this order were (0.18, 0.18), (0.20, 0.20), (0.24, 0.17), (0.19, 0.18), and (0.15, 0.25) for 4X FF, 6X FF, 10X FF, 6X FFF, and 10X FFF, respectively.

**TABLE 1:** The percentage dose differences (average ± s.d.) of the PDDs between the two linacs for different field sizes and energies.

Field size (cm^2^)	4X FF	6X FF	10X FF	6X FFF	10X FFF
3 × 3	−0.05 ± 0.14	−0.06 ± 0.19	−0.18 ± 0.27	0.15 ± 0.10	0.26 ± 0.11
5 × 5	−0.01 ± 0.11	−0.05 ± 0.14	−0.09 ± 0.23	0.13 ± 0.09	0.24 ± 0.15
10 × 10	0.13 ± 0.20	−0.04 ± 0.21	−0.16 ± 0.32	0.09 ± 0.14	0.32 ± 0.15
30 × 30	−0.08 ± 0.16	0.00 ± 0.19	−0.01 ± 0.28	0.03 ± 0.17	0.24 ± 0.15

#### 3.1.2 Output factors

**FIGURE 3:**
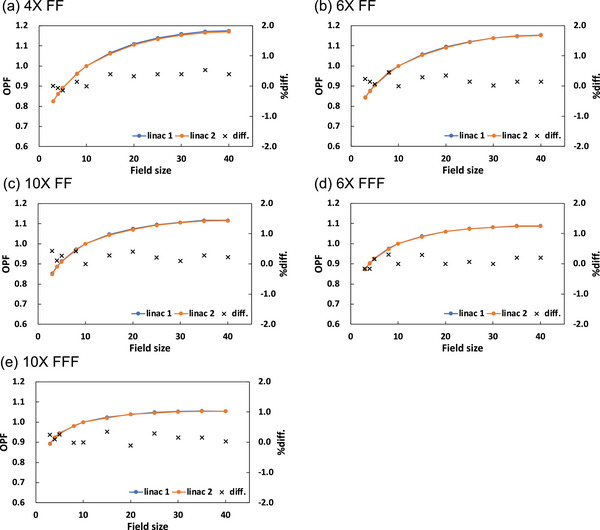
Comparison of measured output factors between the two linacs at each photon energy: (a) 4X FF, (b) 6X FF, (c) 10X FF, (d) 6X FFF, and (e) 10X FFF.

Figure 3a–e compares measured output factors between the two linacs at each photon energy. All DDs were within ±0.5%.

#### 3.1.3 Dose measurement for dynamic MLC plans with constant MLC gaps

**FIGURE 4:**
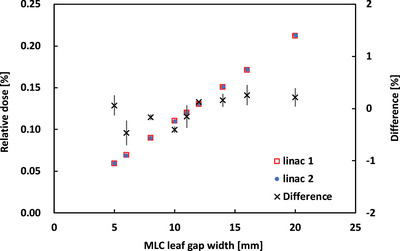
Comparison of relative isocenter doses between the two linacs as a function of the MLC leaf gap width.

The relative doses measured at the two linacs at the isocenter are shown as a function of the MLC leaf gaps in Figure 4. All the measured isocenter DDs between the two linacs were within ±0.5%. In Elekta's MLC alignment, the measurement of the abutting field plan is performed using the iViewGT panel. Therefore, we think that the MLC alignment between the two linacs resulted in a good agreement.

### 3.2 Patient specific QA for VMAT deliveries

Table 2 shows the averages and the ranges of the calculated gamma passing rates (GPRs) under the two different criteria on each linac. All the average GPRs were above 99%, indicating that accurate dose deliveries were achieved. Besides, it was found that the point DDs between the measurement and the calculation by both TPSs using the phantoms were within ±2%, ranging from −1.79% to 1.70%.

**TABLE 2:** Calculated gamma passing rates.

Criteria	linac 1		linac 2	
	3%/2 mm	2%/2 mm	3%/2 mm	2%/2 mm
Ave. GPR (range)	100 (99.5–100)	99.5 (97.6–100)	100 (99.7–100)	99.3 (95.1–100)

### 3.3 Dose distributions and dose difference maps

**FIGURE 5:**
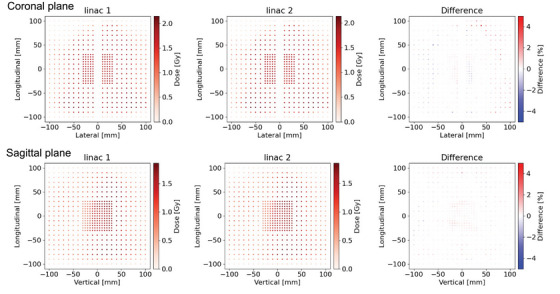
Dose distributions and dose difference maps on coronal and sagittal planes for the head and neck VMAT plan, which was measured by Delta 4.

**FIGURE 6:**
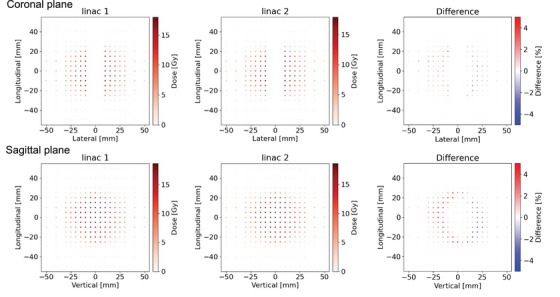
Dose distributions and dose difference maps on coronal and sagittal planes for the liver VMAT plan, which was measured by Delta 4.

**FIGURE 7:**
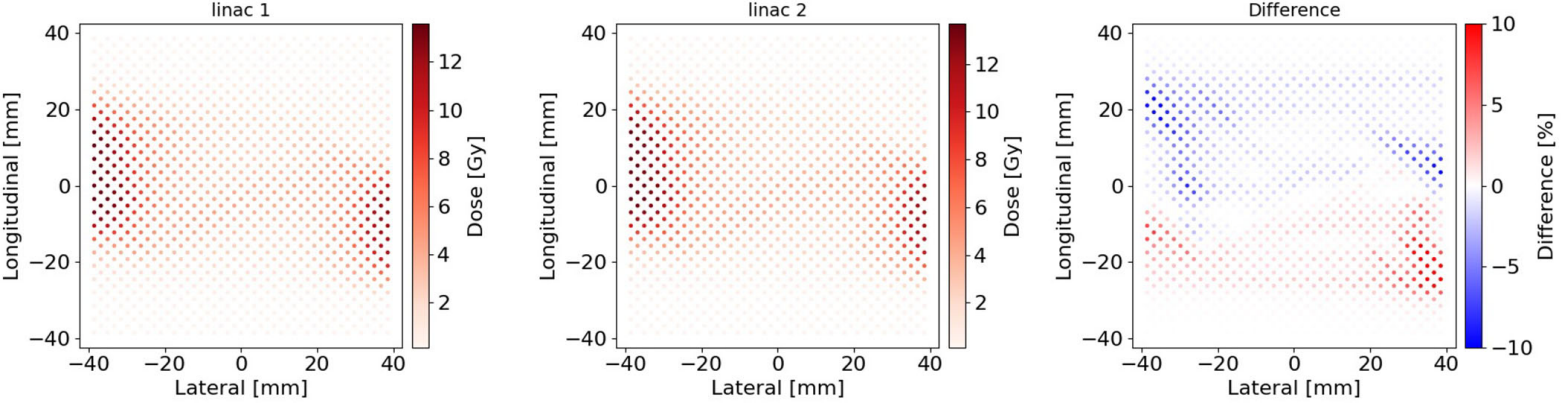
Dose distributions and dose difference maps on coronal plane for the multi‐mets VMAT plan, which was measured by SRS MapCHECK.

**FIGURE 8:**
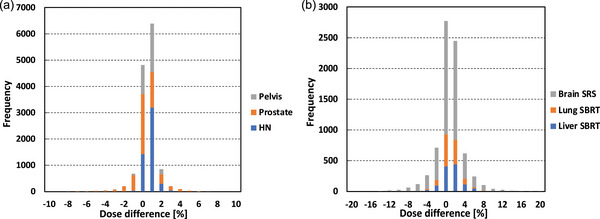
Histograms of dose differences for the different tumor sites (a) with conventional fraction doses for the pelvis, the prostate and the HN VMAT plans, (b) with a single or hypofractionated doses for brain, lung and liver VMAT plans. SRS; stereotactic radiosurgery, SBRT; stereotactic body radiotherapy.

Figures 5, 6, 7 show the dose distributions and DD maps for the VMAT deliveries of the H&N, liver and multiple brain metastases, respectively. Because of the steep dose gradient for the liver SBRT plan and multiple brain metastases SRS plan, a slight misalignment leads to significant dose errors. The histograms of the DDs were shown in Figure 8. The histogram for each tumor site had a peak at a DD of 0%, indicating stable measurement throughout this study. The comparison between the measurement results on the two linacs is shown in Table 3. The average DDs were within 0.25%. For the liver and the multiple brain metastases plans, the standard deviations were larger than the other tumor sites. The differences of these plans are greatly affected by setup errors and detector resolutions. Besides, the average DDs of the point dose measurements were within 0.3%, ranging from −0.19% to 0.30%.

**TABLE 3:** Comparisons of differences (average and s.d.) in dose at each detector measured by the Delta 4 (D4) or the SRS MapCheck (MC) for the different tumor sites between the two linacs. The measured points receiving greater than one‐tenth of the prescribed dose were analyzed. The point doses were also measured and the percent differences between the two linacs were calculated. The TPS employed for each VMAT planning was also indicated. Abbreviations: TPS, treatment planning system, RS, RayStation.

	H&N	Pelvis	Prostate	Lung	Liver	Brain
Dose differences with D4 or MC (%)	0.25 ± 0.54	0.14 ± 0.64	−0.22 ± 1.50	0.00 ± 1.75	0.09 ± 2.29	−0.15 ± 3.18
Point dose differences (%)	0.02 ± 0.22	−0.17 ± 0.51	−0.19 ± 0.31	0.18 ± 0.27	0.30 ± 0.07	N/A
TPS (Case No.)	RS (5)	RS (3)	Monaco (5) /RS (5)	Monaco (2) /RS (2)	RS (3)	RS (2)

## 4 DISCUSSIONS

In this study, we firstly compared the beam properties such as PDDs, output factors, profiles and dynamic MLC dose deliveries with constant MLC gap widths between the two AGL‐matched linacs recently installed in our institution. Subsequently, we compared dosimetric characteristics of VMAT deliveries for different tumor sites between the beam‐matched two linacs. Our findings were that (1) the differences in measurements of PDDs and output factors between the two beam‐matched linacs were less than ±1%, (2) the average gamma indices for inline and crossline profiles between the two matched linacs were less than 0.5, and (3) the relative differences of relative doses in the dynamic MLC plans were ±0.5% between the two linacs. The dose differences of PDD for 10X FFF beams were greater than that for the other beams (Table 1). In the customer acceptance test (CAT), the relative differences between each of the measured PDD10s in the two linacs and the reference PDD10 given by the Elekta were less than 1%. However, the CAT results for the 10X FFF beam were slightly greater than for the other beams. The number of data (two‐linacs data) is not sufficient, and further investigation is required using a more number of AGL‐based linacs. These results indicate that the basic beam properties are clinically identical between the two linacs.

In addition, the GPRs on the two linacs were higher than 99% and 95% using 3%/2 mm and 2%/2 mm gamma criteria, respectively, for all the tumor sites considered in this study. The GPR was obtained by counting the sampling points showing the gamma index of less than one, and therefore it is not sensitive to a high gradient region if the number of the sampling points there is limited. The gamma index is always a positive number showing the relative discrepancy between two dose distributions. For this reason, we calculated the histogram of DD maps and found a peak at a DD of 0%, thereby confirming a good dose agreement between the two linacs.

It was reported that the gamma analysis with vendor‐provided criteria were insufficient for the purpose of beam matching, and stricter gamma criteria were recommended.^4^, ^6^, ^7^, ^8^ To ensure clinically sufficient beam matching, it was insisted that the beam data of each linac should not be matched to that of the reference linac, but the TPS calculation should be used as the reference.^4^ Furthermore, it was recommended that the calculation by TPS beam model may be employed as the reference.^9^ The AGL service adjusts each linac to match the measurement and the beam model in the Monaco TPS, which conforms to the previous recommendations. In this study, we have evaluated the beam properties and dosimetric characteristics for the VMAT delivery, and have confirmed consistency of the dose deliveries including VMAT between the two linacs. The difference between dose distributions for many tumor sites was 0.06 ± 2.12%, and the difference in measured point dose between the two linacs was −0.03 ± 0.33%. The multi‐dimensional detectors were aligned with a room laser. The offsets of 1 mm or less were observed between the measurement with linac 1 and linac 2. To improve the setup accuracy, the translational and rotational shifts could be compensated by HexaPOD couch (Elekta) through the CBCT guidance. But the setup for these detectors typically uses room lasers. The results of this study include a setup error of about 1 mm due to the laser setup. The hot and cold spots may occur in the dose difference map in the case of treatment plan with high gradient dose, therefore, the SD of DD maps is large value. Our result has demonstrated that the tolerance of gamma analysis for the AGL service of 2% DD and 2‐mm DTA is clinically sufficient for VMAT treatments for many tumor sites shown in this study.

## 5 CONCLUSION

To verify the AGL performance, we compared the measured beam data and patient QA results between the two AGL‐matched linacs. Beam characteristics including PDDs, lateral profiles, output factors and relative doses as function of MLC leaf gap widths were in good agreement between two linacs. The differences in measured dose distributions and point doses were 0.06 ± 2.12% and −0.03 ± 0.33%, respectively. It was demonstrated that the AGL service provides an accurate VMAT treatment reproducibility for many tumor sites with gamma pass rates greater than 95% under criteria of 2%/2 mm.

## AUTHOR CONTRIBUTIONS

Masato Tsuneda designed and directed the project, while performing the data collection. He also wrote the manuscript. Kota Abe and Yukio Fujita helped conceive the study and performed the data collection. Ryo Morimoto, Takuma Hashimoto and Yukinao Abe performed the data collection. Takashi Uno reviewed all the clinical VMAT plans. All authors provided critical feedback and helped shape the research to complete the final manuscript.

## CONFLICT OF INTEREST STATEMENT

Masato Tsuneda, Kota Abe and Yukio Fujita receive endowed chairs funded by Elekta K.K.

## Supporting information



Supplementary Information

Supplementary Information

Supplementary Information

Supplementary Information

## References

[1] Chang Z , Wu Q , Adamson J , et al. Commissioning and dosimetric characteristics of TrueBeam system: composite data of three TrueBeam machines. Med Phys. 2012;39(11):6981‐7018. 23127092 10.1118/1.4762682

[2] Tanaka Y , Mizuno H , Akino Y , Isono M , Masai N , Yamamoto T . Do the representative beam data for TrueBeam ™ linear accelerators represent average data? J Appl Clin Med Phys. 2019;20(2):51‐62. 10.1002/acm2.12518 PMC6370991 30636358

[3] Tani K , Wakita A , Tohyama N , et al. Evaluation of differences and dosimetric influences of beam models using golden and multi‐institutional measured beam datasets in radiation treatment planning systems. Med Phys. 2020;47(11):5852‐5871. 32969046 10.1002/mp.14493

[4] Sjöström D , Bjelkengren U , Ottosson W , Behrens CF . A beam‐matching concept for medical linear accelerators. Acta Oncol (Madr). 2009;48(2):192‐200. 10.1080/02841860802258794 18752079

[5] Firmansyah OA , Firmansyah AF , Sunaryati SI , et al. Implementation of beam matching concept for the new installed elekta precise treatment system medical LINACs in Indonesia. Atom Indones. 2021;47(3):181‐189.

[6] Gershkevitsh E , Peraticos A , Dimitriadis Raad D , et al. SU‐GG‐T‐306_ can single dataset in treatment planning system represent several beam‐matched accelerators_ ‐ Gershkevitsh ‐ 2010 ‐ Medical Physics ‐ Wiley Online Library. Med Phys. 2010;37(6Part20):3256‐3256.

[7] Hrbacek J , Depuydt T , Nulens A , Swinnen A , Van Den Heuvel F . Quantitative evaluation of a beam‐matching procedure using one‐dimensional gamma analysis. Med Phys. 2007;34(7):2917‐2927. 17822000 10.1118/1.2745239

[8] Sarkar B , Manikandan A , Nandy M , et al. A mathematical approach to beam matching. Br J Radiol. 2013;86(1031). 10.1259/bjr.20130238 PMC3830429 23995874

[9] Smith K , Balter P , Duhon J , et al. AAPM medical physics practice guideline 8.a.: linear accelerator performance tests. J Appl Clin Med Phys. 2017;18(4):23‐39. 28548315 10.1002/acm2.12080 PMC5874895

