# Supplementary material for: Delivery accuracy of VMAT on two beam‐matched linacs provided by accelerated go live service
Source: J Appl Clin Med Phys. 2023 Jun 16;24(7):e14071. doi: 10.1002/acm2.14071 (PMC10338800; doi:10.1002/acm2.14071)
Supplement: Supplementary file 1 — Supplementary Information [file ACM2-24-e14071-s001.pdf]

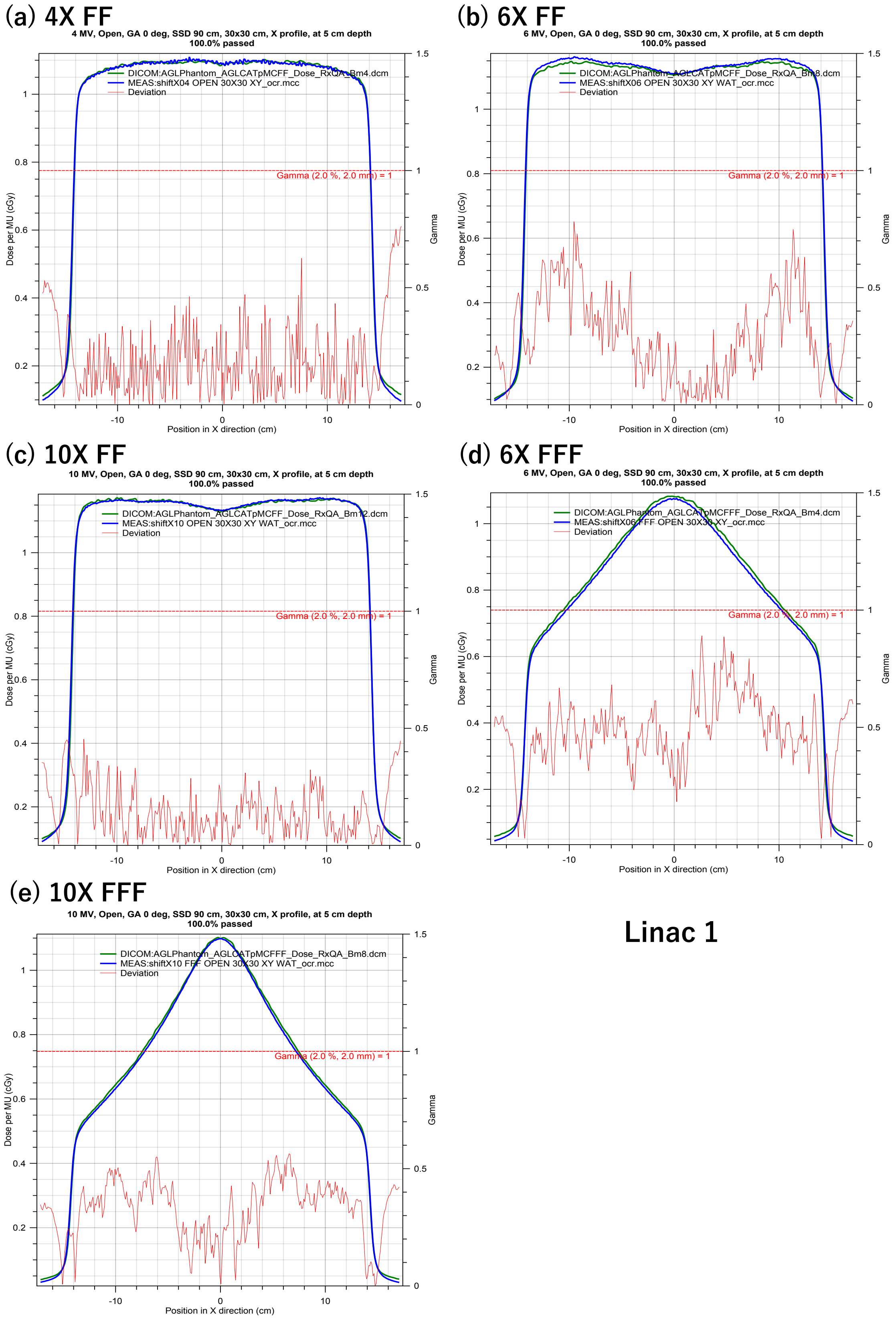
(a) 4X FF
4 MV, Open, GA 0 deg, SSD 90 cm, 30x30 cm, X profile, at 5 cm depth
100.0% passed
DICOM:AGLPhantom_AGLCATpMCFF_Dose_RxQA_Bm4.dcm
MEAS:shiftX04 OPEN 30X30 XY_ocr.mcc
Deviation
Gamma (2.0 %, 2.0 mm) = 1
Dose per MU (cGy)
Gamma
Position in X direction (cm)
(b) 6X FF
6 MV, Open, GA 0 deg, SSD 90 cm, 30x30 cm, X profile, at 5 cm depth
100.0% passed
DICOM:AGLPhantom_AGLCATpMCFF_Dose_RxQA_Bm8.dcm
MEAS:shiftX06 OPEN 30X30 XY WAT_ocr.mcc
Deviation
Gamma (2.0 %, 2.0 mm) = 1
Dose per MU (cGy)
Gamma
Position in X direction (cm)
(c) 10X FF
10 MV, Open, GA 0 deg, SSD 90 cm, 30x30 cm, X profile, at 5 cm depth
100.0% passed
DICOM:AGLPhantom_AGLCATpMCFF_Dose_RxQA_Bm12.dcm
MEAS:shiftX10 OPEN 30X30 XY WAT_ocr.mcc
Deviation
Gamma (2.0 %, 2.0 mm) = 1
Dose per MU (cGy)
Gamma
Position in X direction (cm)
(d) 6X FFF
6 MV, Open, GA 0 deg, SSD 90 cm, 30x30 cm, X profile, at 5 cm depth
100.0% passed
DICOM:AGLPhantom_AGLCATpMCFFF_Dose_RxQA_Bm4.dcm
MEAS:shiftX06 FFF OPEN 30X30 XY_ocr.mcc
Deviation
Gamma (2.0 %, 2.0 mm) = 1
Dose per MU (cGy)
Gamma
Position in X direction (cm)
(e) 10X FFF
10 MV, Open, GA 0 deg, SSD 90 cm, 30x30 cm, X profile, at 5 cm depth
100.0% passed
DICOM:AGLPhantom_AGLCATpMCFFF_Dose_RxQA_Bm8.dcm
MEAS:shiftX10 FFF OPEN 30X30 XY WAT_ocr.mcc
Deviation
Gamma (2.0 %, 2.0 mm) = 1
Dose per MU (cGy)
Gamma
Position in X direction (cm)
Linac 1
