# Supplementary material for: Delivery accuracy of VMAT on two beam‐matched linacs provided by accelerated go live service
Source: J Appl Clin Med Phys. 2023 Jun 16;24(7):e14071. doi: 10.1002/acm2.14071 (PMC10338800; doi:10.1002/acm2.14071)
Supplement: Supplementary file 2 — Supplementary Information [file ACM2-24-e14071-s002.pdf]

(f) 4X FF

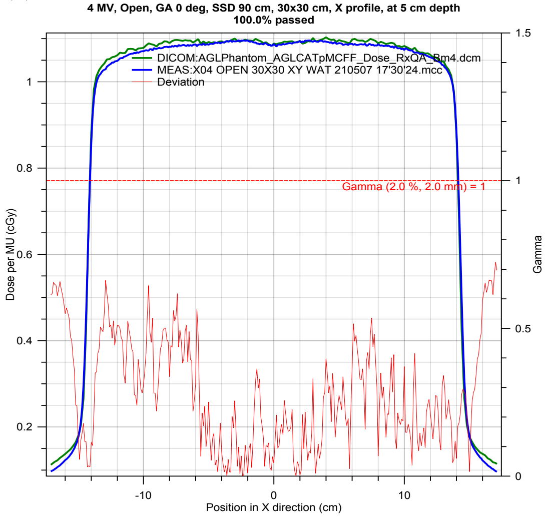

(g) 6X FF

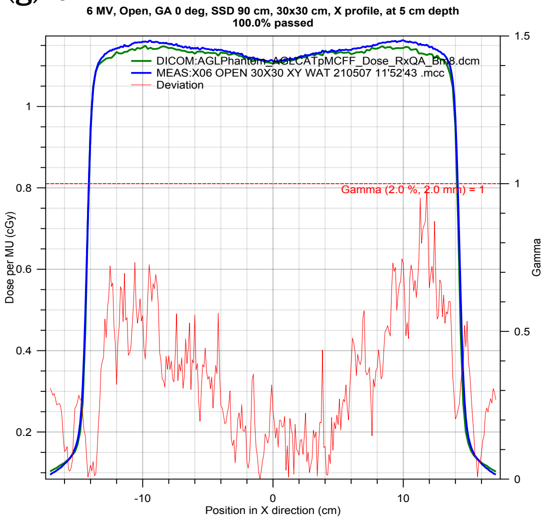

(h) 10X FF

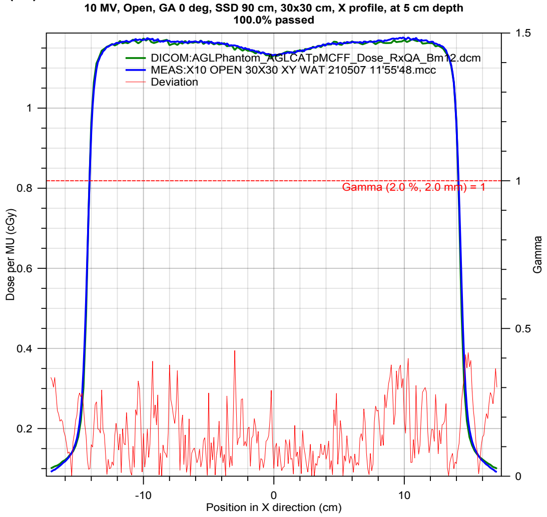

(i) 6X FFF

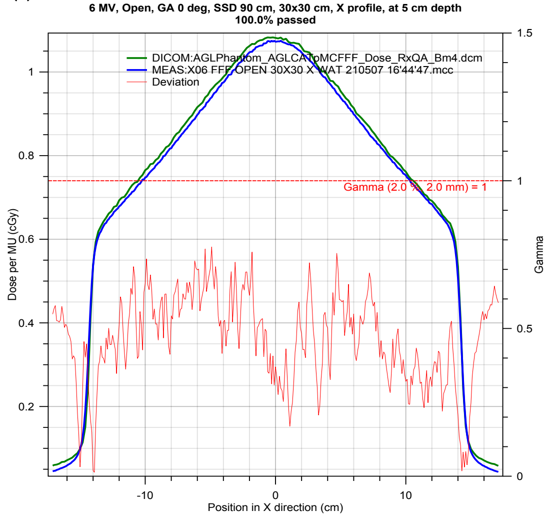

(j) 10X FFF

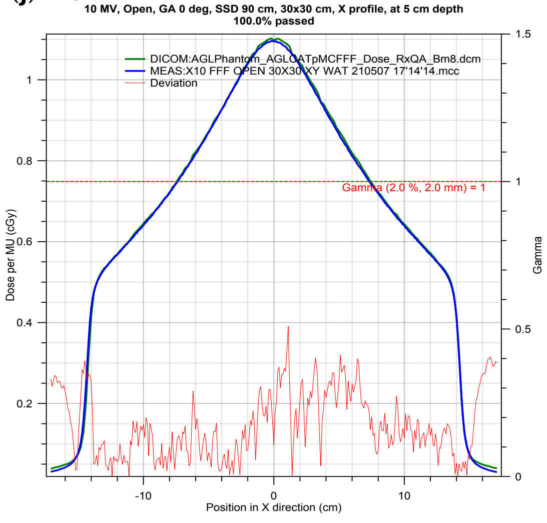

Linac 2
