# Supplementary material for: Delivery accuracy of VMAT on two beam‐matched linacs provided by accelerated go live service
Source: J Appl Clin Med Phys. 2023 Jun 16;24(7):e14071. doi: 10.1002/acm2.14071 (PMC10338800; doi:10.1002/acm2.14071)
Supplement: Supplementary file 3 — Supplementary Information [file ACM2-24-e14071-s003.pdf]

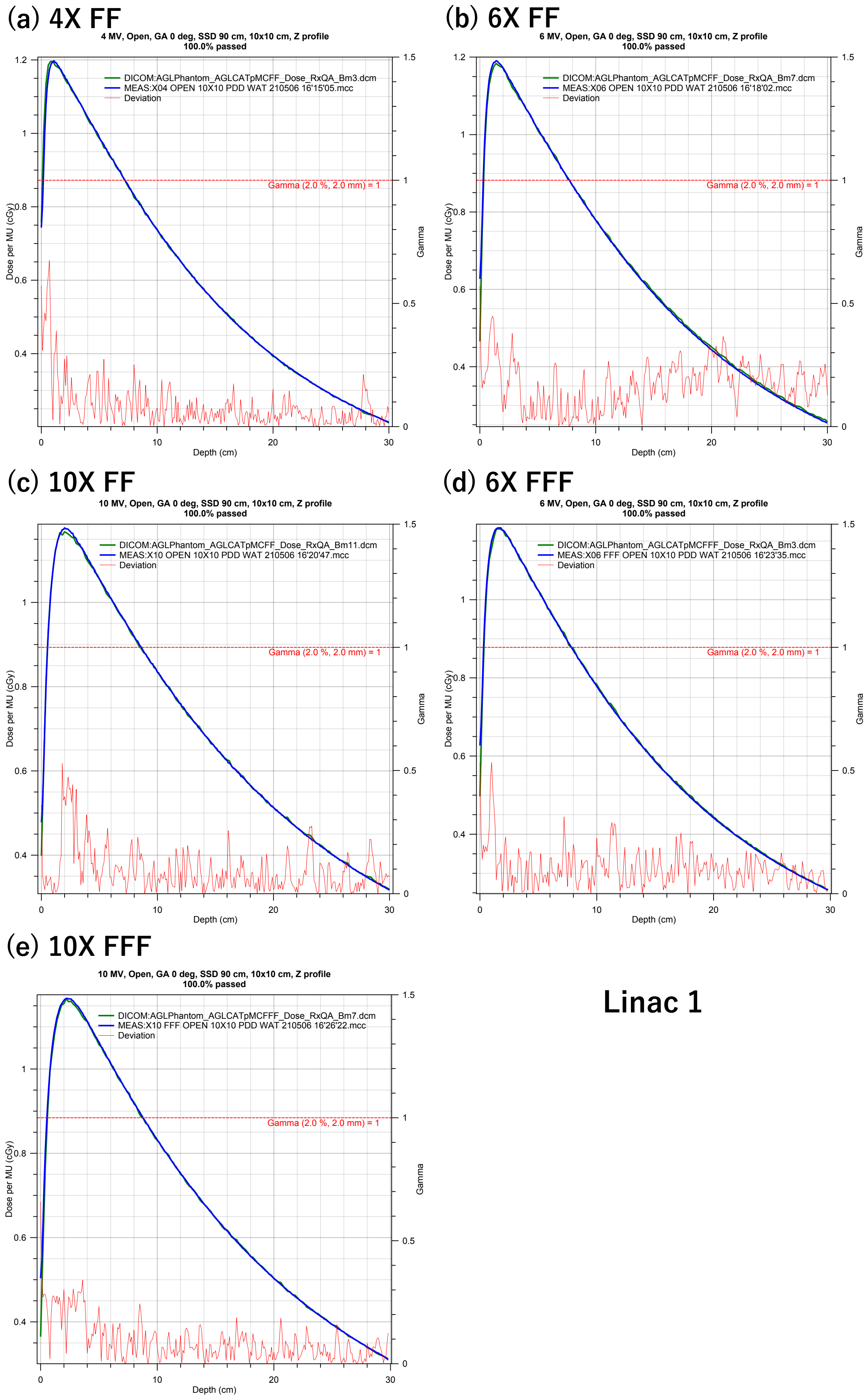
(a) 4X FF
4 MV, Open, GA 0 deg, SSD 90 cm, 10x10 cm, Z profile
100.0% passed
DICOM:AGLPhantom_AGLCATpMCFF_Dose_RxQA_Bm3.dcm
MEAS:X04 OPEN 10X10 PDD WAT 210506 16'15'05.mcc
Deviation
Gamma (2.0 %, 2.0 mm) = 1
Dose per MU (cGy)
Gamma
Depth (cm)
(b) 6X FF
6 MV, Open, GA 0 deg, SSD 90 cm, 10x10 cm, Z profile
100.0% passed
DICOM:AGLPhantom_AGLCATpMCFF_Dose_RxQA_Bm7.dcm
MEAS:X06 OPEN 10X10 PDD WAT 210506 16'18'02.mcc
Deviation
Gamma (2.0 %, 2.0 mm) = 1
Dose per MU (cGy)
Gamma
Depth (cm)
(c) 10X FF
10 MV, Open, GA 0 deg, SSD 90 cm, 10x10 cm, Z profile
100.0% passed
DICOM:AGLPhantom_AGLCATpMCFF_Dose_RxQA_Bm11.dcm
MEAS:X10 OPEN 10X10 PDD WAT 210506 16'20'47.mcc
Deviation
Gamma (2.0 %, 2.0 mm) = 1
Dose per MU (cGy)
Gamma
Depth (cm)
(d) 6X FFF
6 MV, Open, GA 0 deg, SSD 90 cm, 10x10 cm, Z profile
100.0% passed
DICOM:AGLPhantom_AGLCATpMCFFF_Dose_RxQA_Bm3.dcm
MEAS:X06 FFF OPEN 10X10 PDD WAT 210506 16'23'35.mcc
Deviation
Gamma (2.0 %, 2.0 mm) = 1
Dose per MU (cGy)
Gamma
Depth (cm)
(e) 10X FFF
10 MV, Open, GA 0 deg, SSD 90 cm, 10x10 cm, Z profile
100.0% passed
DICOM:AGLPhantom_AGLCATpMCFFF_Dose_RxQA_Bm7.dcm
MEAS:X10 FFF OPEN 10X10 PDD WAT 210506 16'26'22.mcc
Deviation
Gamma (2.0 %, 2.0 mm) = 1
Dose per MU (cGy)
Gamma
Depth (cm)
Linac 1
