# Supplementary material for: Delivery accuracy of VMAT on two beam‐matched linacs provided by accelerated go live service
Source: J Appl Clin Med Phys. 2023 Jun 16;24(7):e14071. doi: 10.1002/acm2.14071 (PMC10338800; doi:10.1002/acm2.14071)
Supplement: Supplementary file 4 — Supplementary Information [file ACM2-24-e14071-s004.pdf]

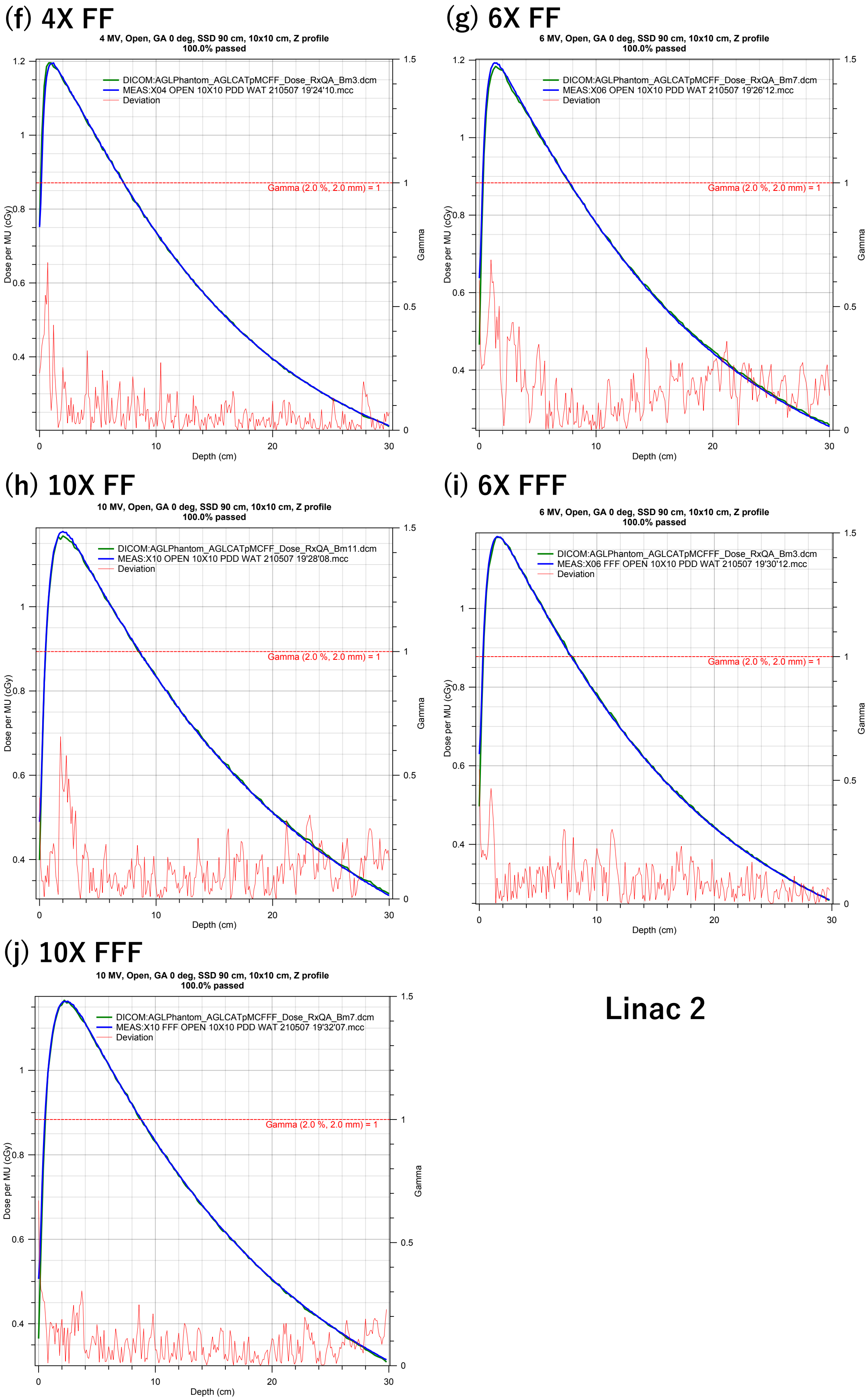
(f) 4X FF
4 MV, Open, GA 0 deg, SSD 90 cm, 10x10 cm, Z profile
100.0% passed
DICOM:AGLPhantom_AGLCATpMCFF_Dose_RxQA_Bm3.dcm
MEAS:X04 OPEN 10X10 PDD WAT 210507 19'24'10.mcc
Deviation
Gamma (2.0 %, 2.0 mm) = 1
Dose per MU (cGy)
Gamma
Depth (cm)
(g) 6X FF
6 MV, Open, GA 0 deg, SSD 90 cm, 10x10 cm, Z profile
100.0% passed
DICOM:AGLPhantom_AGLCATpMCFF_Dose_RxQA_Bm7.dcm
MEAS:X06 OPEN 10X10 PDD WAT 210507 19'26'12.mcc
Deviation
Gamma (2.0 %, 2.0 mm) = 1
Dose per MU (cGy)
Gamma
Depth (cm)
(h) 10X FF
10 MV, Open, GA 0 deg, SSD 90 cm, 10x10 cm, Z profile
100.0% passed
DICOM:AGLPhantom_AGLCATpMCFF_Dose_RxQA_Bm11.dcm
MEAS:X10 OPEN 10X10 PDD WAT 210507 19'28'08.mcc
Deviation
Gamma (2.0 %, 2.0 mm) = 1
Dose per MU (cGy)
Gamma
Depth (cm)
(i) 6X FFF
6 MV, Open, GA 0 deg, SSD 90 cm, 10x10 cm, Z profile
100.0% passed
DICOM:AGLPhantom_AGLCATpMCFFF_Dose_RxQA_Bm3.dcm
MEAS:X06 FFF OPEN 10X10 PDD WAT 210507 19'30'12.mcc
Deviation
Gamma (2.0 %, 2.0 mm) = 1
Dose per MU (cGy)
Gamma
Depth (cm)
(j) 10X FFF
10 MV, Open, GA 0 deg, SSD 90 cm, 10x10 cm, Z profile
100.0% passed
DICOM:AGLPhantom_AGLCATpMCFFF_Dose_RxQA_Bm7.dcm
MEAS:X10 FFF OPEN 10X10 PDD WAT 210507 19'32'07.mcc
Deviation
Gamma (2.0 %, 2.0 mm) = 1
Dose per MU (cGy)
Gamma
Depth (cm)
Linac 2
